# Retained Intra-uterine Foetal Bones Resulting in Secondary Infertility: A Case Report

**DOI:** 10.7759/cureus.2575

**Published:** 2018-05-03

**Authors:** Nitu Mishra, Rekha Bharti, Pratima Mittal, Jyotsna Suri, Divya Pandey

**Affiliations:** 1 Department of Obstetrics and Gynaecology, VMMC and Safdarjung Hospital, New Delhi, IND

**Keywords:** abortion, infertility, fetal bones, hysteroscopy

## Abstract

Termination of pregnancy (abortion) is a reasonably safe procedure when properly performed. However, even after being performed with due precautions, complications may occur. Retention of fetal products following a mid-trimester abortion is a rare complication of abortion which can then result in secondary infertility. We are here presenting the case of a young woman who underwent an abortion in the 15th week of gestation due to anencephaly in the fetus and who then failed to conceive for the next five years. Transvaginal ultrasonographic examination suggested the presence of bone-like structures in the uterine cavity which were removed hysteroscopically. The patient was able to conceive after the hysteroscopic removal of bony fragments. Retained bony fragments from a previous mid-trimester abortion should thus be kept in the list of differentials of secondary infertility.

## Introduction

The advances in medicine both from a perspective of diagnosis and treatment have made the event of conceiving and childbirth safer. The ability to terminate or abort a pregnancy for foetal defects spares the unborn child and the family from hardships which would have been the fate in case the child was born. Even though the procedure of termination of a pregnancy is safe [1], it can at times result in unwanted immediate complications like haemorrhage, uterine perforation, cervical injury and at times in late complications like infections, bleeding, menstrual abnormalities and uterine synechiae. As 50% of patients undergoing medical termination are young adults, there is a possibility of secondary infertility due to an abortion which has become complicated [2].

Occasionally if the abortion is done in the second trimester or if the foetus was removed by destructive means, some parts of the foetus may inadvertently be left behind in the uterine cavity. In such a scenario, there might be pelvic pain, abnormal bleeding, infection, dyspareunia or even passage of foetal bones per vaginum [3]. Retained foetal bones may even lead to secondary infertility. Few reports have been published describing secondary infertility after abortion. It is expected that retained foetal bones would induce a uterine reaction in the form of discharge per vaginum and/or pain in the majority of such patients while also leading to secondary infertility.

We are presenting the case of a young female who had secondary infertility without any other associated complaints. She had a previous second-trimester abortion five years back secondary to foetal indications. And while she had returned to her usual activities, she had failed to conceive again.

## Case presentation

A 28-year-old woman was referred to the Gynaecology out-patient clinic of Safdarjung Hospital for evaluation and management of infertility. Detailed history was obtained which suggested that she was having secondary infertility. She had delivered a healthy baby boy seven years back. Two years after the birth of the first child, she conceived again. However during the second pregnancy, at 15 weeks of gestation, the foetus was found to have anencephaly on ultrasonographic examination. A decision for termination of the pregnancy was made and induction of labour was done. However, the induction failed and the treating obstetrician removed the foetus per vaginally in piece-meal. Following the abortion, her menstrual cycles returned to normal and she had no other complaints. But she could not conceive again after the abortion. Over the period of the past five years, she consulted multiple physicians and received numerous treatments, but was unsuccessful in conceiving. She was then referred to our hospital.

**Figure 1:**
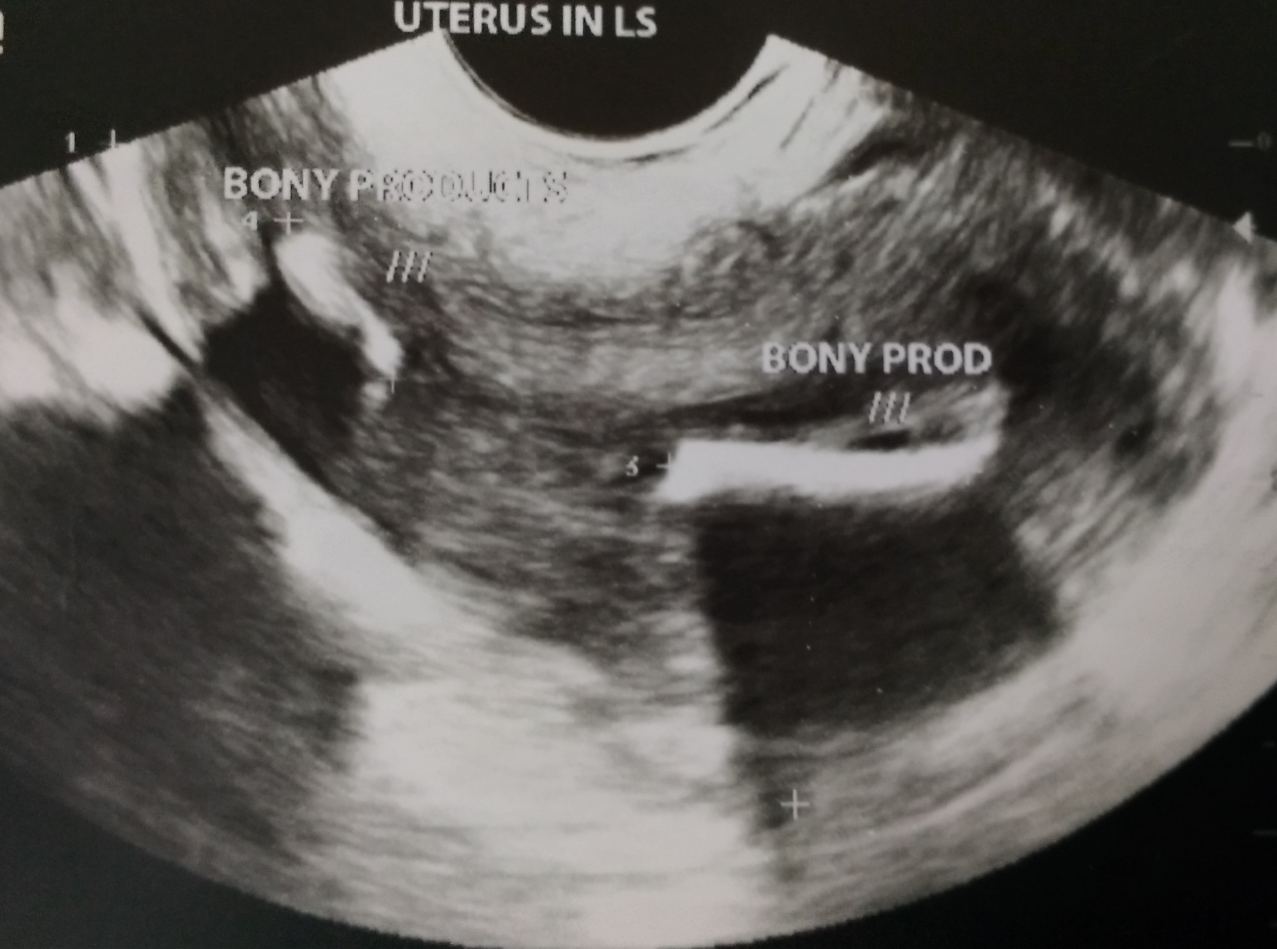
Trans-vaginal ultrasonographic image showing echogenic structures suggestive of bone-like structure.

**Figure 2:**
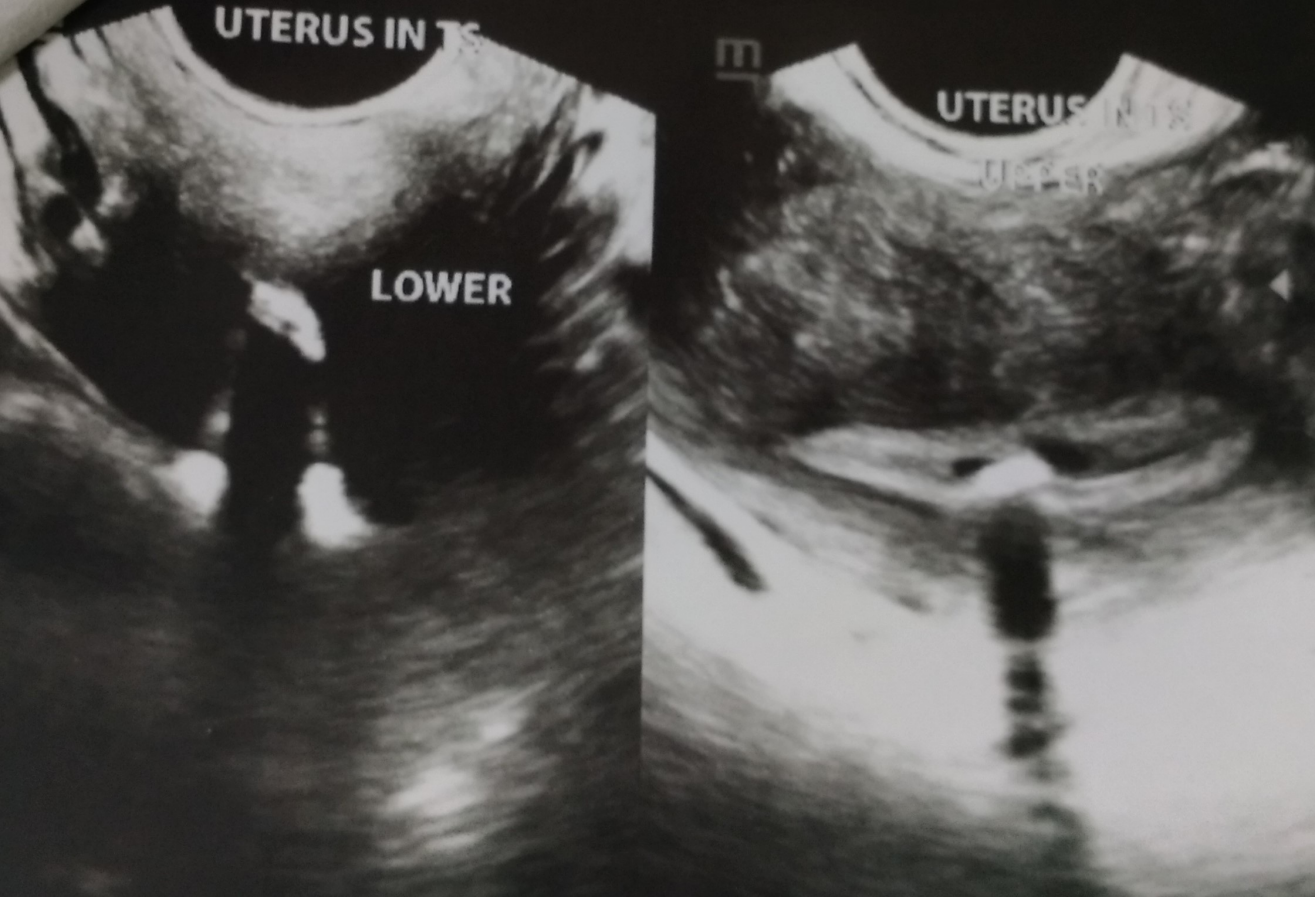
Trans-vaginal ultrasonographic images at different levels.

On physical examination, the patient had an average built. Her vitals were within normal range. Pelvic examination was done per vaginally and per abdominally, both of which were unremarkable. We performed a transvaginal ultrasonographic evaluation of the uterus and the genital tract. Multiple echogenic areas were found in the uterine cavity suggestive of bone-like structures/foreign bodies in the uterus (Figures 1, 2). The findings were confirmed by the consultant radiologist of our hospital. Hysteroscopy and removal of the bony products was planned.

**Figure 3:**
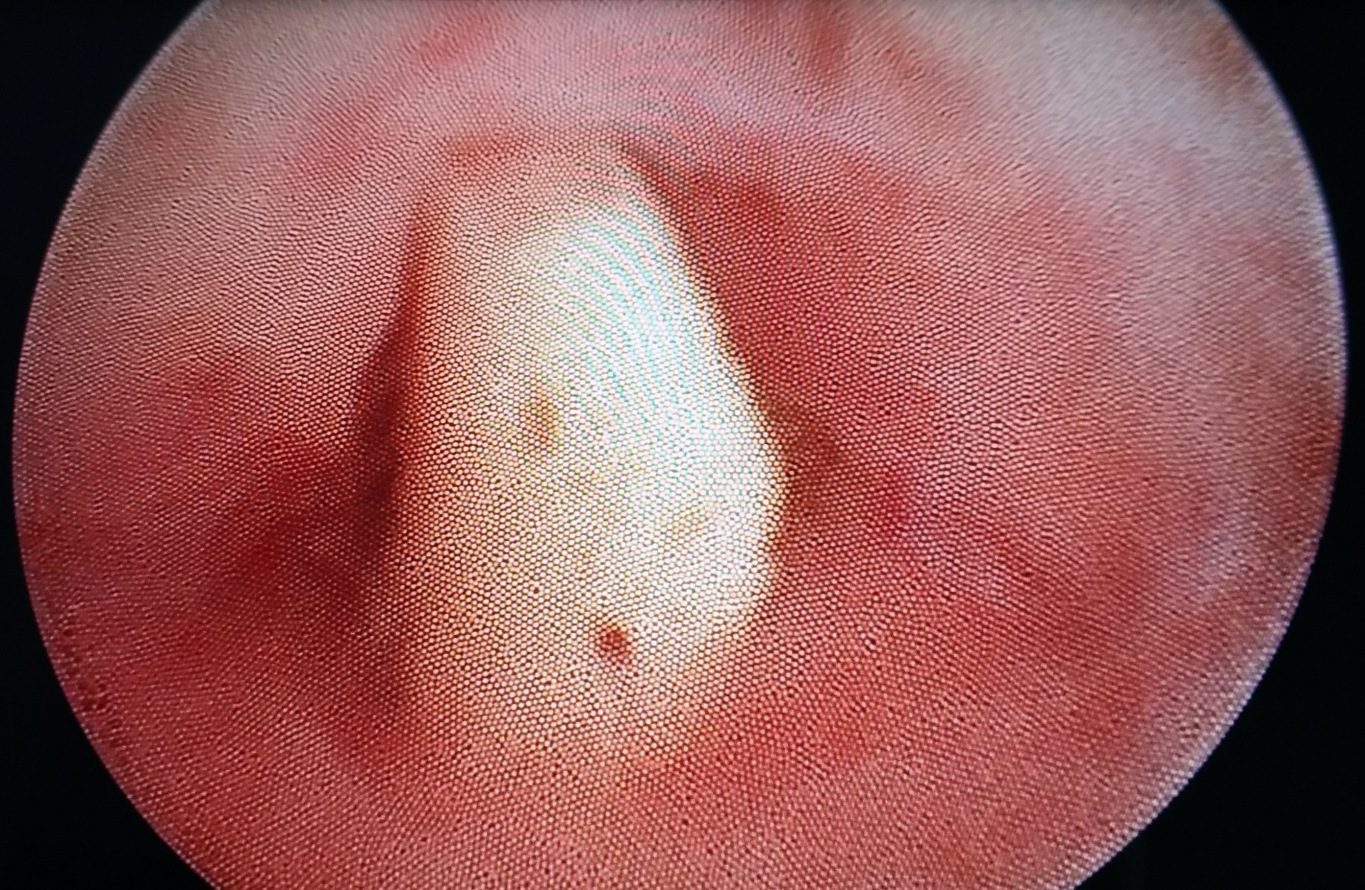
Hysteroscopic image showing the whitish colored bony structure.

**Figure 4:**
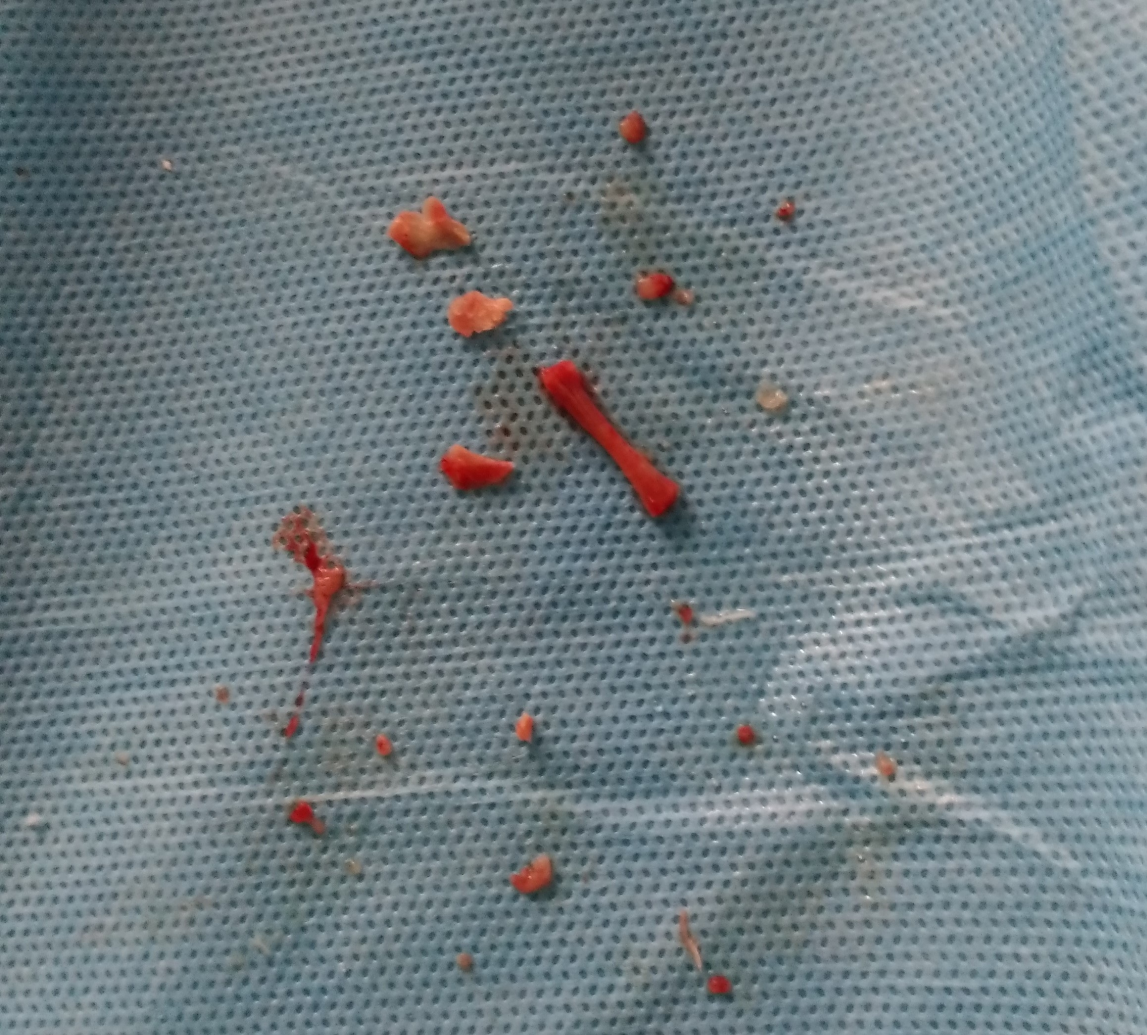
Image showing all the bony products removed from the uterine cavity.

Under general anaesthesia, diagnostic hysteroscopy was performed which showed the presence of a cylindrical structure resembling a miniature long bone (Figure 3) and multiple small fragments in the uterus. We removed the long bony structure along with few of the relatively large-sized fragments under vision. The smaller fragments could not be removed individually, and dilation and curettage was performed to remove the fragments. Overall, around seven reasonably large-sized and numerous other small bony pieces were retrieved from the patient’s uterus (Figure 4). In the post hysteroscopy period, the patient was kept under cover of intravenous antibiotics for 24 hours. All the extracted material was sent for histopathological examination and was reported by the pathologist as bone.

First follow-up was done after her first menses. Subsequent follow-ups were done at monthly intervals. Her menstrual cycle was not altered after the procedure and she was able to conceive again five months from the hysteroscopic removal of bone fragments. Thus, confirming our assumption of secondary infertility due to the retained foetal bony fragments.

## Discussion

While termination of pregnancy (abortion) is a safe procedure when properly performed, it has its own complications. Incomplete evacuation of the uterus can result in multiple complications including the rare complication of secondary infertility as presented by us in this case. A number of hypotheses have been offered in the literature attempting to explain the possible reason for infertility in such cases. One of the hypotheses proposes that retained foetal tissues from a previous incomplete abortion can stimulate dystrophic calcification and lead to an ossification of the foetal tissue thereby resulting in bone formation [4]. Chronic inflammation and tissue destruction have also been postulated to stimulate heterotopic bone formation in mature uterine stroma [5]. Both the scenarios are highly unlikely in the case presented here. The extraction of a relatively large tubular bone resembling a long bone of foetus was indicative of its origin from being from a foetus and not because of dystrophic or heterotrophic calcification. Also, the histopathologic examination of the extracted fragments confirmed the diagnosis. Previously published case reports are suggestive that retained foetal bones may be responsible for secondary infertility [2, 6-8].

Retained foetal bone has been thought to act as an intrauterine contraceptive device altering the uterine milieu and preventing implantation [8,9]. Other hypotheses as to why retained foetal bones lead to infertility include prevention of implantation because of obliteration of the uterine cavity and toxicity of bone particles on an implanted embryo [10].

Whatever the etiology might be, obtaining a thorough history is a must while evaluating a patient of secondary infertility. In case a history of previous abortion is provided, the obstetrician should keep in mind the remote possibility of retained foetal bones as a cause of infertility. Trans-vaginal ultrasonography must be done in all cases and in case any suspicious echoes are seen hysteroscopy should be planned.

Hysteroscopic removal of the bony pieces followed by curettage should be done in a patient with retained foetal bones as it has been proven in available case reports to be a sufficient treatment of such infertility. In the present case too, after removal of the bone pieces, the menses resumed, and the patient conceived in five months’ time.

Considering that most reports of retention of foetal bones have been in patients’ with second-trimester abortions, a suggestion of routine trans-vaginal ultrasound to evaluate the completeness of evacuation can be made.

We believe that the present report adds to the pool of knowledge of practising obstetricians and makes them aware about considering retained bones from previous abortion in the list of differentials of secondary infertility.

## Conclusions

Secondary infertility due to retained foetal bones is a rare condition which an obstetrician should keep in mind while evaluating such patients with a prior history of mid-trimester abortion. Trans-vaginal ultrasound is an effective modality for initial diagnosis. Hysteroscopy can be used for both confirmation and treatment. Fertility is expected to return for most of such patients. All patients with second-trimester abortion should have a trans-vaginal ultrasound in the post-abortion period to confirm complete evacuation of all the products of conception.
